# Genetic Diversity and Ecological Evaluation of Fluorescent Pseudomonads Isolated from the Leaves and Roots of Potato Plants

**DOI:** 10.1264/jsme2.ME11237

**Published:** 2012-02-22

**Authors:** Nobutaka Someya, Tomohiro Morohoshi, Tsukasa Ikeda, Kenichi Tsuchiya, Seishi Ikeda

**Affiliations:** 1 Hokkaido Agricultural Research Center, National Agriculture and Food Research Organization, 9–4 Shinsei-minami, Memuro-cho, Kasai-gun, Hokkaido 082–0081, Japan; 2 Department of Material and Environmental Chemistry, Graduate School of Engineering, Utsunomiya University, 7–1–2 Yoto, Utsunomiya, Tochigi 321–8585, Japan; 3 Faculty of Agriculture, Kyushu University, Hakozaki, Higashi-ku, Fukuoka 812–8581, Japan

**Keywords:** *Pseudomonas*, potato, 16S rRNA, leaf, root

## Abstract

A total of 828 isolates of fluorescent pseudomonads (FPs) were obtained from the leaves (305 isolates) and roots (523 isolates) of potato plants grown in different geographical locations in Japan, and 16S rRNA gene sequences of 776 isolates were successfully determined by direct PCR sequencing. Clustering analysis (≥99% identity) identified 13 and 26 operational taxonomic units (OTUs) for leaf- and root-associated FPs, respectively, and 29 OTUs were identified in the phytosphere of potato plants. Among them, 7 and 9 OTUs showed a significantly biased distribution to the leaves and roots, respectively. Phylogenetic analysis revealed that 3 dominant OTUs for leaf-associated FPs were grouped in a cluster of leaf-associated pathogens, such as *Pseudomonas cichorii* and *Pseudomonas viridiflava*. In contrast, 4 OTUs were located in a cluster of saprophytic pseudomonads. Among them, 3 OTUs showed high similarity to *Pseudomonas koreensis* and *Pseudomonas vancouverensis*, both of which have been reported to be beneficial for biological control or plant growth promotion. These data provide key information for efficient surveying and utilization of beneficial FPs in agricultural practices.

Potato (*Solanum tuberosum* L.) is one of the world’s most important crops (35). In Japan, most commercial potato cultivars are susceptible to a range of phytopathogens and require repeated fungicide applications for the control of diseases. Repeated fungicide applications may result in fungicide resistance, soil contamination, or harm to non-target organisms, which may have important ecological roles (35). Therefore, the development of an alternative disease management strategy that is environmentally friendly and inexpensive is desired as a part of the development of sustainable agricultural systems. The biological control of plant diseases using antagonistic microbes is such an alternative. Biological control utilizes the natural beneficial functions of microbes; therefore, a better understanding of their ecological characteristics under agricultural conditions is essential.

The genus *Pseudomonas* consists of 128 species (24). Most *Pseudomonas* species are saprophytic inhabitants of water- and soil-related environments. Some strains of *Pseudomonas* spp., in particular fluorescent pseudomonads (FPs), are widely known to be beneficial to plants as biological control agents of plant diseases or as plant growth-promoting agents (1, 2, 4, 5, 9, 14). FP is a generic term employed to describe bacteria exhibiting characteristics similar to the genus *Pseudomonas*. Although there have been a number of studies on the use of beneficial FPs as biological control agents in controlled environments, their practical application to plant disease control has been reported only rarely (10). This is most likely because their beneficial effects are unstable under field conditions. In the field, biotic and abiotic environmental factors positively or negatively affect the diversity and functionality of beneficial FPs in plant disease control. Therefore, an understanding of the ecological traits of plant-associated microbes, such as their tissue specificity or stability in diverse environments, is essential for consistent and effective use of biological control agents under practical agronomic circumstances. Indeed, it has been reported that the tissue colonization and biological control activity of FPs are greatly affected by various biotic and abiotic environmental factors (26). In particular, several studies have demonstrated that the genetic composition of FPs was considerably different among the rhizospheres of different plant species cultivated in the same soil (3, 18). Our previous study suggested the presence of different taxonomic groups of pseudomonads between the leaves and roots of potato plants (31).

As a result of recent advances in sequencing technologies and bioinformatics, sequence-based community analysis is now a powerful tool for providing unambiguous ecological information considering both species richness and abundance. Such ecological assessments provide very useful data for the efficient screening of beneficial microbes and for reliable and consistent application of microbiological agents under field conditions, as pointed out by Picard and Bosco (25). Complex interactions between various environmental factors and FP communities can now be resolved by mass sequence analyses in conjunction with relatively simple bioinformatics. It has been reported that genetic information obtained with the 16S rRNA gene region generally correlates to the diversity based on the whole genome of FPs (13, 20, 27); thus, analysis of the 16S rRNA gene would be an efficient way of evaluating the genetic diversity of FPs.

In the present study, community analysis based on 16S rRNA gene sequencing was conducted on FPs isolated from the leaves and roots of potato plants grown in diverse geographic locations in Japan. The results clarified the diversity, tissue specificity, and geographic heterogeneity of FPs in potato plants, and it will facilitate the application of beneficial FPs in agricultural practices.

## Materials and Methods

### Sampling of plant tissues and isolation of FPs

A total of 123 plant samples were collected from commercial potato fields in diverse locations in Japan (Table 1). The sampling locations ranged from a latitude of 44°17′ N and longitude of 144°35′ E (Hokkaido) to a latitude of 31°3′ N and longitude of 130°44′ E (Kagoshima). Both the leaf and root tissues were harvested at the flowering stage. Visible soil particles were removed from the root samples with sterile tweezers.

Leaf or root tissues (1 g) were dipped in 9 mL sterile 15 mM phosphate buffer (pH 7.0). Samples were sonicated with a ULTRASONIC WASHER (USM-1; SND, Nagano, Japan) at 42 kHz for 1 min to release the microbial cells attached to the leaves or roots. Serial dilutions of the cells were cultivated on King’s B medium agar (hereafter termed KBA) containing 50 μg mL^−1^ cycloheximide by incubating at 25°C in the dark for 3 days. FPs could be distinguished from the other bacterial colonies when subjected to UV irradiation. Five fluorescent colonies were randomly selected for each sample.

### Sequence analysis of 16S rRNA genes

Each bacterial isolate was inoculated into 2 mL Luria Bertani (LB) liquid medium (Sigma-Aldrich Japan, Tokyo, Japan) and incubated for 24 h at 25°C on a reciprocal shaker (140 rpm). The bacterial cells were centrifuged, and the total bacterial genomic DNA was extracted using the DNeasy blood and tissue kits (Qiagen, Hilden, Germany) according to the manufacturer’s instructions. Genomic DNA obtained from the bacterial isolates was amplified by polymerase chain reaction (PCR) performed in an iCycler system (Bio-Rad, Hercules, CA, USA) with the following primers; 27F (5′-AGAGTTTGATCMTGGCTCAG-3′) and 1525R (5′-AAGGAGGT GWTCCARCC-3′), which targeted a 16S rRNA gene fragment of approximately 1.5 kb. The total volume of the reaction mixture was 25 μL; the mixture contained 12.5 μL of Premix Taq (Takara Bio, Otsu, Japan), 0.5 μM of each primer, 50–100 ng bacterial genomic DNA, and sterile distilled water. The thermal cycling program was as follows: initial denaturation at 94°C for 3 min; followed by 30 cycles of 94°C for 30 s, 55°C for 30 s, and 72°C for 1 min, and final extension at 72°C for 10 min.

The PCR products were separated by electrophoresis performed on 1.5% agarose gels using Tris-acetate ethylenediaminetetraacetic acid (TAE) as a buffer (Bio-Rad) at 100 V for 20 min. The gels were stained with ethidium bromide for 20 min and subsequently rinsed in sterile water. Thereafter, the PCR products were visualized with a transilluminator (FAS-III; Toyobo, Osaka, Japan). The expected size of an amplicon was confirmed by comparison with a 1 kb DNA ladder (Bayou Biolabs, Harahan, LA, USA).

One-pass sequencing was conducted for the 16S rRNA gene using 27F primer by the Takara Dragon Genomic Center (Takara Bio). Sequences were manually edited to eliminate primer and low-quality region sequences. The sequences were analyzed for the orientation and detection of non-16S rDNA sequences using OrientationChecker (http://www.bioinformatics-toolkit.org/Squirrel/index.html). Subsequently, the 5′-region of the 16S rRNA gene (corresponding to bases 109–665 of the *Escherichia coli* 16S rRNA gene) was used for sequence analyses.

Sequences were placed in a taxonomic hierarchy using the Classifier in Ribosomal Database Project (RDP) II (34), and the sequences of non-*Pseudomonas* species were eliminated. The sequences were then aligned using CLUSTAL_X (32) and the alignments were written in a PHYLogeny Inference Package (PHYLIP) format file. Using the information from this file, distance matrices were constructed using the DNADIST program with default parameters (7). The resulting matrices were used as an input to the software MOTHUR (30), which was used to generate diversity indices and richness indicators and to conduct clustering analysis. The operational taxonomic units (OTUs) in the clustering analysis were defined by 99% sequence identity. A series of diversity indexes (Cao1, ACE, Shannon and Simpson) were calculated (12, 16, 30). A type strain of the closest known species to a representative sequence of an OTU was retrieved using the SeqMatch in RDP II (34). The representative sequences of OTUs were aligned using CLUSTAL_X, and were used to build a phylogenetic tree by the neighbor-joining (NJ) method (28) with type strains of known species. The topology of the constructed tree was evaluated by bootstrap analysis with 1,000 replicates (6). The trees were constructed using TreeView software (23).

### Accession numbers of nucleotide sequences

Nucleotide sequences of the partial 16S rRNA genes of FPs isolated from leaves and roots have been deposited in the DNA Data Bank of Japan (DDBJ) under accession numbers AB628216–AB628491 and AB628492–AB628991, respectively.

## Results

### FP bacteria isolated from leaves and roots of potato

When the KBA was used for bacterial isolation, approximately 10^3^–10^5^ colony-forming units (CFU) (leaf) and 10^5^–10^7^ CFU (root) of bacterial colonies were obtained from 1 g (fresh weight) of potato leaves and roots, respectively, for most of the samples. Fluorescent colonies were not observed from some of the leaf samples. As a result, 305 isolates were obtained from leaves, while 523 isolates were obtained from the roots (Table 1).

### Statistical analyses of diversity of potato-associated FPs

A total of 828 isolates of FPs were obtained from potato plants collected in various locations in Japan. As a result of direct PCR sequencing of the 16S rRNA gene, 276 and 500 sequences were successfully determined for leaf and root isolates, respectively (Table 2). Using the RDP Classifier, all of the isolates were confirmed to belong to the genus *Pseudomonas*. The numbers of OTUs observed for leaf- and root-associated FPs were 13 and 26, respectively. Library coverage was more than 99% for both leaf- and root-associated FPs. The Chao1 and abundance-based coverage estimator (ACE) values for root-associated FPs were higher than those for leaf-associated FPs. On the other hand, the Shannon and Simpson indexes were shown to be similar between leaf- and root-associated FPs.

### Spatial distribution of potato-associated FPs

**Fig. 1:**
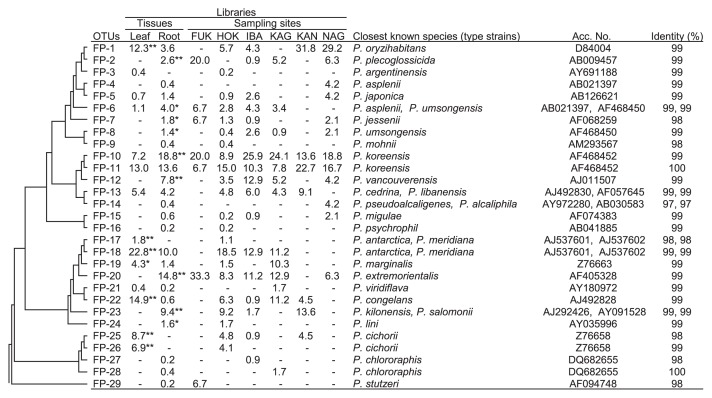
Phylogenetic distribution of operational taxonomic units (OTUs) of fluorescent pseudomonads (FPs) isolated from the phyllosphere and rhizosphere of potato plants cultivated in various locations across Japan. The dendrogram indicates the phylogenetic relationships among the representative sequences of OTUs (defined by ≥99% identity). The table indicates the relative abundance (%) of clones belonging to each OTU in each column and the results of a pair-wise BLAST between a representative sequence and its closest type strain. The sampling sites (prefecture) were as follows: FUK, Fukuoka; HOK, Hokkaido; IBA, Ibaraki; KAG, Kagoshima; KAN, Kanagawa; NAG, Nagasaki. The percentages of isolates analyzed are shown for each column of libraries. “*” and “**” mean statistical significance at 0.05 and 0.01 levels, respectively.

Clustering analysis (>99% identity) revealed the presence of 29 OTUs for FPs in potato plants (Fig. 1). Among them, a significantly biased distribution of the numbers of isolates to the leaves compared with the roots was observed for 7 OTUs (FP-1, 17, 18, 19, 22, 25, and 26) and 9 OTUs (FP-2, 6, 7, 8, 10, 12, 20, 23, and 24), respectively. Meanwhile, 2 dominant OTUs (FP-11 and 13) were shown to be evenly present in both leaves and roots. Regarding the geographic distribution, most of the dominant OTUs (FP-1, 10, 11, 12, 13, 18, 20, 22, and 23) were present in a wide range of geographic locations in Japan. Among them, 2 OTUs in particular (FP-10 and 11) appeared to be ubiquitously present across Japan.

### Phylogenetic diversity of potato-associated FPs

**Fig. 2:**
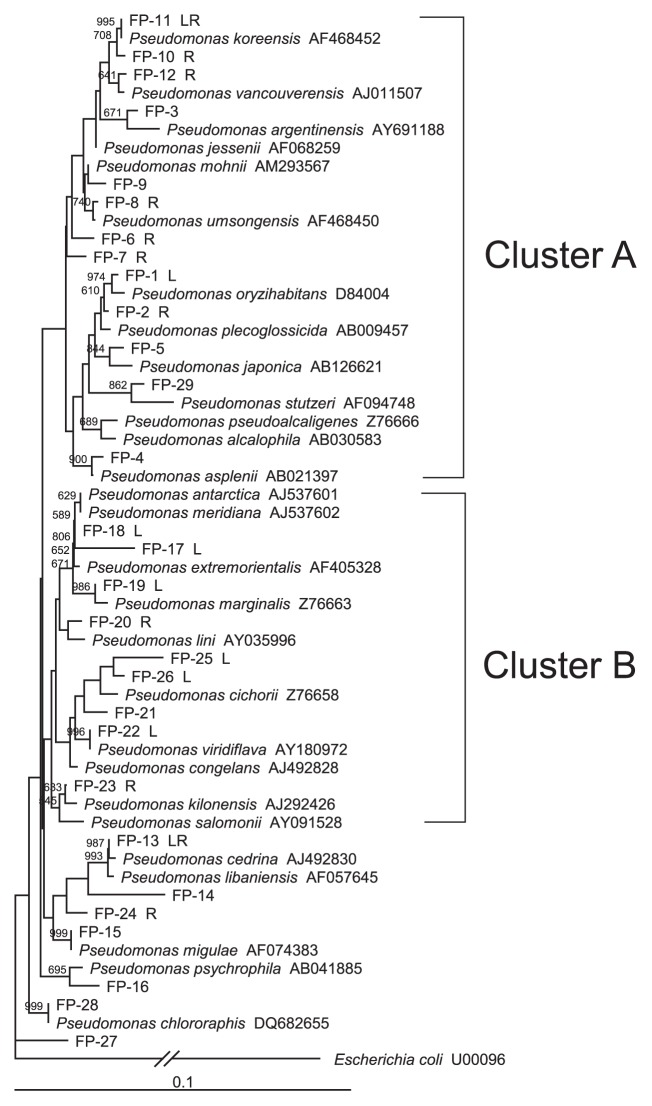
Phylogenetic tree of 16S rRNA genes based on the representative sequences of operational taxonomic units (OTUs) for potato-associated fluorescent pseudomonads (FPs). The tree was constructed using the neighbor-joining method. Scale represents 0.1 substitutions per site. Numbers at the nodes are the proportions of 1,000 bootstrap resamplings, and values <500 are not shown. Dominant OTUs showing a significantly biased distribution in terms of the numbers of isolates in the leaf compared with the root are indicated as L and R, respectively. LR indicates dominant OTUs with no significantly biased distribution to the leaf or root.

Analysis with SeqMatch in RDP retrieved 28 sequences of type strains of *Pseudomonas* species as the closest known species to the representative sequences (Fig. 1). Pair-wise BLAST analyses showed that most of the representative sequences of the dominant OTUs had high identity (99% or 100%) to known species, except OTU FP-25, a dominant OTU showing distribution specific to leaves (8.7%; Fig. 1), and had an identity of only 98% to *P. cichorii*. On the other hand, several representative sequences of minor OTUs (FP-7, 9, 14, 17, 27, and 29) showed low identity (97–98%) to known species. Phylogenetic analysis revealed no distinct relationship between the overall phylogenetic positions of OTUs and their tissue specificity (Fig. 2); however, there was a certain degree of correlation between the phylogenetic location of some OTUs and the numbers of isolates distributed preferentially to leaves or roots. The pathogenic pseudomonads to human (*Pseudomonas oryzihabitans* and *Pseudomonas stutzeri*), animals (*Pseudomonas plecoglossicida* and *P. stutzeri*), and plants (*Pseudomonas pseudoalcaligenes*, *Pseudomonas asplenii*, *Pseudomonas marginalis*, *Pseudomonas cichorii*, and *Pseudomonas viridiflava*) have been reported (24). Cluster A in Figure 3 represents the phylogenetic positions of most of the OTUs, which showed a biased distribution to roots. In contrast, the majority of dominant OTUs that showed a biased distribution to leaves were located in cluster B (Fig. 2).

## Discussion

A total of 828 isolates of FPs were collected from potato plants cultivated in diverse locations in Japan. Statistical analyses, shown by the library coverage values in Table 2, suggested that the numbers of samples taken for both leaves and roots were high enough to survey the diversity of FPs under the examined conditions. As expected, the number of OTUs for roots (23 OTUs) was more than that for leaves (13 OTUs); however, interestingly, both the Shannon and Simpson indexes showed similar values for both leaf- and root-associated FPs, indicating that similar community structures are shared between leaf- and root-associated FPs, regardless of marked environmental differences among them.

Clustering analysis (≥99% identity) revealed the presence of 29 OTUs for FPs in potato plants (Fig. 1). Detailed phylogenetic analysis indicated that the genetic diversity of potato-associated FPs was widespread within the entire genus *Pseudomonas* (Fig. S1). Among the dominant OTUs, isolates for 4 OTUs (FP-1, 22, 25, and 26) and 3 OTUs (FP-12, 20, and 23) were exclusively obtained from leaves and roots, respectively, suggesting the presence of tissue specificity for colonization in some FPs. OTU FP-1 was shown to be closely related to *Pseudomonas oryzihabitans*, which was originally isolated from rice paddy soil in Japan (15). *P. oryzihabitans* is not known as an epiphytic colonizer, but has been reported as an opportunistic pathogen (8) and is also known for its nematicidal effects (29). Phylogenetic analyses revealed that OTUs FP-22, 25, and 26 are closely related to a series of leaf-associated pathogenic pseudomonads in a cluster (Figs. 2 and S1). OTU FP-12 was found to be closely related to *P. vancouverensis*, which was isolated from forest soil (22) and was recently reported to be a plant growth-promoting bacterium (21). Meanwhile, 4 OTUs (FP-10, 11, 13, and 18) were recognized as dominant OTUs for both leaves and roots (Fig. 1). In detailed phylogenetic analyses, 3 OTUs (FP-10, 11, and 13) were positioned in a cluster that included FP-12 (Fig. S1). OTUs FP-10 and 11 showed a close relationship with *P. koreensis*, which was originally isolated from agricultural soil in Korea (17). Recently, Hultberg *et al.*(11) demonstrated that a strain of *Pseudomonas koreensis* had the ability to control late blight on potato. Furthermore, isolates in these 3 OTUs (FP-10, 11, and 13) were stably detected from various cultivars grown in diverse locations in Japan (Table S1). Considering their high persistence in the environment, these 3 OTUs (FP-10, 11, and 13) could be a good resource for surveying a candidate microbe for biological control; however, these OTUs, widely distributed in the potato phytosphere, may consist of several genotypes that were not detected under the experimental conditions used in the present study. The whole genome-based method should be applied to examine the genetic diversity of these dominant OTUs in future studies. Tissue specificity for colonization of the isolates also needs to be examined in future work through an inoculation test.

Although the biocontrol characteristics of FPs are not clearly explained by the phylogenetic relationships at present (10), detailed analyses of the genetic diversity of FPs would facilitate the evaluation of the relationships between certain phylotypes of beneficial microbes and host plants under various environmental conditions as shown in the studies of Mavrodi *et al.*(19). In addition, as pointed out by Hultberg *et al.*(11), the use of nonpathogenic microorganisms is a fundamental and essential issue for biological control. Among *Pseudomonas*, 23 and 16 species have been shown to exhibit pathogenicity against plants and animals (including humans), respectively (10). Appropriate evaluation of the genetic diversity of FPs would minimize the risk of unwanted effects of FPs on the environment. For example, the detailed phylogenetic analyses conducted in the present study revealed that 4 OTUs (FP-21, 22, 25, and 26) were included in a cluster of leaf-associated pathogens, which included *P. cichorii* and *P. viridiflava* (cluster A in Fig. S1). The isolates in these OTUs could be harmful to plants based on this phylogenetic position. Indeed, the isolates in OTU FP-25 have been shown to have a deleterious effect on potato leaves (31). In contrast, 4 OTUs (FP-10, 11, 12, and 13) were located in a cluster of saprophytic pseudomonads (cluster B in Fig. S1). Among them, 3 OTUs showed high similarity to *P. koreensis* and *P. vancouverensis*, both of which have been reported to be beneficial pseudomonads. These results suggest that the phylogenetic position of FPs could partially imply their biological features.

In conclusion, the present study revealed the tissue specificity and geographic distribution of the dominant groups of FPs in the phytosphere of potato plants. Through clustering analyses, defined as >99% identity, 4 and 3 dominant OTUs were shown to be exclusively present in the leaf and root tissues, respectively. Three dominant OTUs were present in both leaves and roots, 2 of which were shown to be distributed in a wide range of geographic locations in Japan. These results will allow the efficient survey of beneficial microbes and provide a new strategy for biological control from ecological viewpoints.

## Supplementary material



## Figures and Tables

**Table 1:** Metadata of samples analyzed in the present study

Sampling sites	Season (month/year)	No. of fields	No. of plant samples (No. of cultivars)	No. of isolates^a^	

				Leaf	Root
Fukuoka (FUK)	Oct/2008	1	4 (1)	0	15
Hokkaido (HOK)	Aug–Oct/2008–2009	24	67 (13+α)^b^	201	286
Ibaraki (IBA)	May–Jun/2006–2009	9	19 (4)	35	84
Kagoshima (KAG)	Feb/2009	3	16 (1)	48	74
Kanagawa (KAN)	Jun/2009	1	3 (2)	15	15
Nagasaki (NAG)	Oct/2008	5	14 (1)	6	49
Total		43	123 (18+α)	305	523

a Five colonies were randomly isolated from each sample. Less than 5 or no fluorescent colonies were observed in some samples.

b +α indicates local lines of potato.

**Table 2:** Statistical summary of genetic diversity of potato-associated fluorescent pseudomonads

Libraries	Leaf	Root
Statistics		
No. of sequences	276	500
OTUs^a^	13	23
No. of singletons	1	3
Library coverage (%)^b^	99.6	99.4
Diversity indexes		
Chao1	13	23.6
ACE	10.1	24.9
Shannon index (*H*′)	2.1	2.3
Simpson index (1/*D*)	6.4	6.4

a OTUs were defined at 99% sequence identity.

b Cx = 1 − (*n*/*N*), where *nx* is the number of singletons that are encouraged only once in a library and *N* is the total number of clones.
