# Supplementary material for: Genetic Diversity and Ecological Evaluation of Fluorescent Pseudomonads Isolated from the Leaves and Roots of Potato Plants
Source: Microbes Environ. 2012 Feb 22;27(2):122–6. doi: 10.1264/jsme2.ME11237 (PMC4036014; doi:10.1264/jsme2.ME11237)
Supplement: Supplementary file 1 [file 27_122_s1.pdf]

Figure S1 legends

Fig. S1. Phylogenetic tree of 16S rRNA genes based on the representative sequences of operational taxonomic units (OTUs) for potato-associated fluorescent pseudomonads (FPs). The tree was constructed using the neighbor-joining method. The scale represents 0.1 substitutions per site. The numbers at the nodes are the proportions of 1,000 bootstrap resamplings, and values of <500 are not shown.

Someya et al.

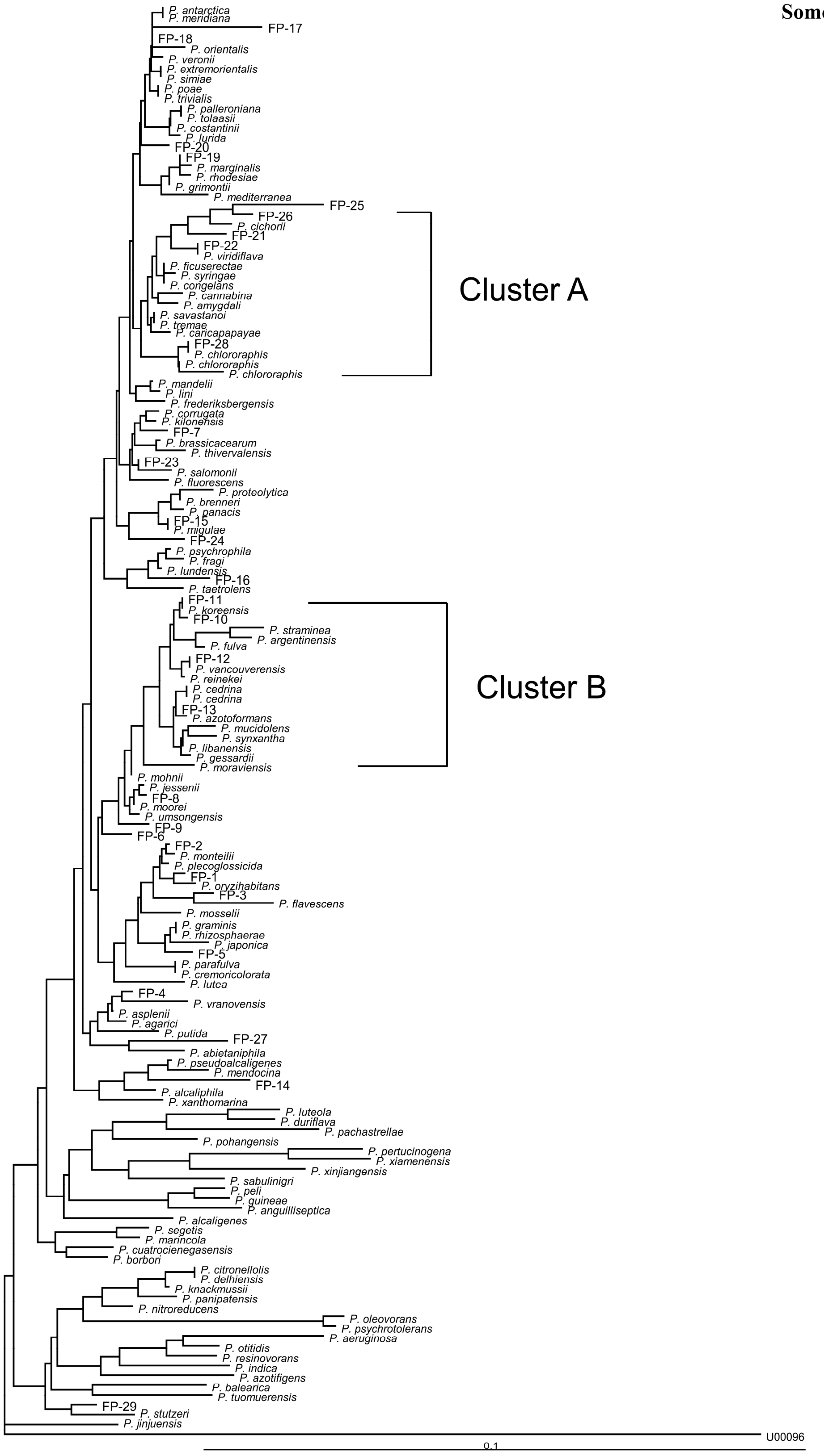

Fig. S1.

Table S1. Distribution of relative abundance of isolates for OTUs defined at 99% identity for potato-associated fluorescent pseudomonad

	Libraries																													
	Tissues		Sites[a]						Varieties[b]																					
OTUs	Leaf	Root	FUK	HOK	IBA	KAG	KAN	NAG	BEN	DAN	DEJ	HAN	HOK	INK	KTA	KTH	KOG	KON	LOC	MAY	NIS	NOR	SAS	SAY	SNO	TOY	WAS	Closest known species	Acc. No.	Identity (%)
FP-1	2.3**	3.6	0.0	5.7	4.3	0.0	31.8	29.2	31.8	1.5	0.0	0.0	40.0	4.3	0.0	20.0	0.0	6.3	4.1	3.1	8.5	0.0	40.0	0.0	11.8	15.4	13.5	*P. putida*	EF204247	100
FP-2	0.0	2.6**	20.0	0.0	0.9	5.2	0.0	6.3	0.0	1.5	20.0	0.0	0.0	0.0	0.0	0.0	0.0	0.0	0.0	0.0	5.5	0.0	0.0	0.0	0.0	0.0	0.0	*P. putida*	HQ166061	100
FP-3	0.4	0.0	0.0	0.2	0.0	0.0	0.0	0.0	0.0	0.0	0.0	0.0	0.0	0.0	0.0	0.0	0.0	0.0	0.0	3.1	0.0	0.0	0.0	0.0	0.0	0.0	0.0	*P. fulva*	EU594555	99
FP-4	0.0	0.4	0.0	0.0	0.0	0.0	0.0	4.2	0.0	0.0	0.0	0.0	0.0	0.0	0.0	0.0	0.0	0.0	0.0	0.0	1.2	0.0	0.0	0.0	0.0	0.0	0.0	*P putida*	FJ010624	100
FP-5	0.7	1.4	0.0	0.9	2.6	0.0	0.0	4.2	0.0	0.0	0.0	0.0	0.0	0.0	0.3	0.0	0.0	0.0	0.5	6.3	1.2	0.0	0.0	0.0	0.0	3.8	0.0	*P. putida*	EF204244	100
FP-6	1.1	4.0*	6.7	2.8	4.3	3.4	0.0	0.0	0.0	1.5	6.7	0.0	0.0	8.7	0.0	0.0	7.4	18.8	1.8	12.5	2.4	0.0	0.0	0.0	5.9	0.0	2.7	*P. fluorescens*	AM293678	99
FP-7	0.0	1.8*	6.7	1.3	0.9	0.0	0.0	2.1	0.0	1.5	6.7	0.0	0.0	0.0	0.0	0.0	18.5	0.0	0.5	0.0	0.6	0.0	0.0	0.0	0.0	0.0	0.0	*P. putida*	FN313522	100
FP-8	0.0	1.4*	0.0	0.4	2.6	0.9	0.0	2.1	0.0	0.0	0.0	0.0	0.0	0.0	0.2	0.0	3.7	0.0	0.5	0.0	1.2	0.0	0.0	0.0	0.0	0.0	2.7	*P. putida*	AY973267	100
FP-9	0.0	0.4	0.0	0.4	0.0	0.0	0.0	0.0	0.0	0.0	0.0	0.0	0.0	0.0	0.0	0.0	3.7	0.0	0.0	0.0	0.0	0.0	0.0	2.4	0.0	0.0	0.0	*P. putida*	D85995	99
FP-10	7.2	8.8**	20.0	8.9	25.9	24.1	13.6	18.8	13.6	29.4	20.0	0.0	0.0	17.4	0.0	0.0	3.7	6.3	10.1	18.8	22.6	0.0	0.0	11.9	11.8	0.0	27.0	*P. fluorescens*	HQ166099	100
FP-11	13.0	13.6	6.7	15.0	10.3	7.8	22.7	16.7	22.7	17.6	6.7	23.5	20.0	43.5	0.2	0.0	0.0	18.8	11.0	9.4	10.4	4.8	0.0	19.0	11.8	26.9	8.1	*P. putida*	EF204240	100
FP-12	0.0	7.8**	0.0	3.5	12.9	5.2	0.0	4.2	0.0	13.2	0.0	0.0	0.0	0.0	0.2	0.0	18.5	0.0	2.8	0.0	4.9	0.0	0.0	11.9	0.0	0.0	10.8	*P. putida*	DQ232745	100
FP-13	5.4	4.2	0.0	4.8	6.0	4.3	9.1	0.0	9.1	10.3	0.0	0.0	30.0	0.0	0.0	0.0	3.7	6.3	5.5	9.4	3.0	0.0	0.0	0.0	5.9	3.8	0.0	*P. synxantha*	EF204250	100
FP-14	0.0	0.4	0.0	0.0	0.0	0.0	0.0	4.2	0.0	0.0	0.0	0.0	0.0	0.0	0.0	0.0	0.0	0.0	0.0	0.0	1.2	0.0	0.0	0.0	0.0	0.0	0.0	*P gessardii*	AY972280	97
FP-15	0.0	0.6	0.0	0.2	0.9	0.0	0.0	2.1	0.0	1.5	0.0	0.0	0.0	0.0	0.0	0.0	3.7	0.0	0.0	0.0	0.6	0.0	0.0	0.0	0.0	0.0	0.0	*P. migulae*	HQ202819	99
FP-16	0.0	0.2	0.0	0.2	0.0	0.0	0.0	0.0	0.0	0.0	0.0	0.0	0.0	0.0	0.0	0.0	0.0	0.0	0.0	0.0	0.0	0.0	0.0	2.4	0.0	0.0	0.0	*P. fragi*	GU549487	99
FP-17	1.8*	0.0	0.0	1.1	0.0	0.0	0.0	0.0	0.0	0.0	0.0	0.0	0.0	0.0	0.0	0.0	0.0	0.0	2.3	0.0	0.0	0.0	0.0	0.0	0.0	0.0	0.0	*P. marginalis*	GQ845121	98
FP-18	[illegible]2.8**	10.0	0.0	18.5	12.9	11.2	0.0	0.0	0.0	8.8	0.0	47.1	10.0	8.7	0.2	60.0	0.0	6.3	21.6	12.5	7.9	4.8	0.0	14.3	23.5	26.9	21.6	*P. marginalis*	GQ845121	99
FP-19	4.3*	1.4	0.0	1.5	0.0	10.3	0.0	0.0	0.0	1.5	0.0	0.0	0.0	4.3	0.0	0.0	0.0	18.8	0.9	0.0	7.3	0.0	0.0	0.0	0.0	0.0	0.0	*P. marginalis*	AY071916	100
FP-20	0.0	4.8**	33.3	8.3	11.2	12.9	0.0	6.3	0.0	11.8	33.3	0.0	0.0	0.0	0.0	0.0	0.0	0.0	14.7	6.3	11.0	9.5	0.0	0.0	23.5	0.0	8.1	*P. frederiksbergensis*	FJ796428	100
FP-21	0.4	0.2	0.0	0.0	0.0	1.7	0.0	0.0	0.0	0.0	0.0	0.0	0.0	0.0	0.0	0.0	0.0	0.0	0.0	0.0	1.2	0.0	0.0	0.0	0.0	0.0	0.0	*P. viridiflava*	Z76671	98
FP-22	4.9**	0.6	0.0	6.3	0.9	11.2	4.5	0.0	4.5	0.0	0.0	0.0	0.0	8.7	0.0	0.0	11.1	0.0	5.0	0.0	7.9	38.1	0.0	14.3	0.0	0.0	0.0	*P. viridiflava*	Z76671	100
FP-23	0.0	9.4**	0.0	9.2	1.7	0.0	13.6	0.0	13.6	0.0	0.0	0.0	0.0	0.0	0.0	0.0	22.2	18.8	13.3	3.1	0.0	0.0	0.0	11.9	0.0	0.0	0.0	*P. mandelii*	DQ377771	99
FP-24	0.0	1.6*	0.0	1.7	0.0	0.0	0.0	0.0	0.0	0.0	0.0	0.0	0.0	4.3	0.0	0.0	0.0	0.0	0.0	0.0	0.0	33.3	0.0	0.0	0.0	0.0	0.0	*P. putida*	EF413074	98
FP-25	8.7**	0.0	0.0	4.8	0.9	0.0	4.5	0.0	4.5	0.0	0.0	11.8	0.0	0.0	0.0	20.0	0.0	0.0	2.3	12.5	0.0	0.0	60.0	0.0	5.9	23.1	2.7	*P. cichorii*	Z76658	97
FP-26	6.9**	0.0	0.0	4.1	0.0	0.0	0.0	0.0	0.0	0.0	0.0	17.6	0.0	0.0	0.0	0.0	3.7	0.0	3.2	3.1	0.0	9.5	0.0	11.9	0.0	0.0	0.0	*P. cichorii*	Z76658	98
FP-27	0.0	0.2	0.0	0.0	0.9	0.0	0.0	0.0	0.0	0.0	0.0	0.0	0.0	0.0	0.0	0.0	0.0	0.0	0.0	0.0	0.0	0.0	0.0	0.0	0.0	0.0	2.7	*P. fluorescens*	AY947533	100
FP-28	0.0	0.4	0.0	0.0	0.0	1.7	0.0	0.0	0.0	0.0	0.0	0.0	0.0	0.0	0.0	0.0	0.0	0.0	0.0	0.0	1.2	0.0	0.0	0.0	0.0	0.0	0.0	*P. chlororaphis*	HM241942	100
FP-29	0.0	0.2	6.7	0.0	0.0	0.0	0.0	0.0	0.0	0.0	6.7	0.0	0.0	0.0	0.0	0.0	0.0	0.0	0.0	0.0	0.0	0.0	0.0	0.0	0.0	0.0	0.0	*P. monteilii*	EF600892	100
No.[c]	276	500	15	459	116	116	22	48	22	68	15	17	10	23	11	5	27	16	218	32	164	21	5	42	17	26	37			

[a]Sampling sites are abbreviated as following; FUK, Fukuoka; HOK, Hokkaido; IBA, Ibaraki; KAG, Kagoshima; KAN, Kanagawa; NAG, Nagasaki.

[b]Varieties are abbreviated as follows; BEN, Beniazuma; DAN, Danshaku; DEJ, Dejima; HAN, Hanashibetsu; HOK, Hokkaikogane; INK, Inkanohitomi; KTA, Kitaakari; KTH, Kitahime; KOG, Koganemaru; KON, Konafubuki; LOC, Local lines; MAY, Mayqueen; NIS, Nishiyutaka; NOR, Norin No. 1; SAS, Sashii; SAY, Sayaakane; SNO, Snowden; TOY, Toyoshiro; WAS, Waseshiro.

[c]Numbers of isolates analyzed for each column.
